# Therapeutic effects of a 45S5 bioactive glass–modified self-etch adhesive on sound and artificially demineralized dentin: mechanical, interfacial, and chemical analyses

**DOI:** 10.1007/s10266-026-01326-2

**Published:** 2026-02-05

**Authors:** Ana Laura Mendes Mota, Marcelo Victor Sidou Lemos, Mateus Soares de Araújo, Victoria Patrício de Oliveira Costa, Juliano Sartori Mendonça, Sérgio Lima Santiago

**Affiliations:** 1 Graduate Program in Dentistry, Faculty of Pharmacy, Dentistry and Nursing, Federal University of Ceará, Fortaleza, Ceará Brazil; 2 Department of Operative Dentistry, Faculty of Pharmacy, Dentistry and Nursing, Federal University of Ceará, Rua Monsenhor Furtado s/n, Fortaleza, Ceará 60430-655 Brazil

**Keywords:** Dentin, Remineralization, Bioglass, Bioactivity

## Abstract

To evaluate the effects on the bonding interface and remineralization potential of a self-etch adhesive containing 10% (w/v) 45S5 bioactive glass (BAG) in sound dentin (SD) and artificially demineralized dentin (ADD). Sound third molars were selected and stored in 0.01% thymol solution, and part of them was subjected to a pH-cycling protocol to induce controlled demineralization. The experimental groups were: SD, ADD, BAG + SD, and BAG + ADD. Microtensile bond strength (n = 8) was assessed after 24 h and six months. Nanoleakage (n = 3), micropermeability (n = 3), Knoop microhardness (n = 3 after 24 h and three months), and FT-IR spectroscopy (n = 3 immediately and after 30 days) were also performed. All specimens were stored in artificial saliva at 37 °C with monthly renewal. Data were analyzed using ANOVA and Tukey’s test (p < 0.05). The BAG + ADD group maintained bond strength after 6 months (p = 0.887). BAG reduced nanoleakage in both SD and ADD. Micropermeability showed no fluorescein uptake in SD, and reduced absorption in ADD when treated with BAG. After 3 months, BAG + ADD significantly increased microhardness at all tested depths, while BAG + SD showed improvements at 70 µm and 100 µm (p = 0.006 and p = 0.015, respectively) FT-IR analysis showed phosphate and carbonate bands in BAG-containing adhesives, with increased definition after 30 days, indicating time-dependent ion release and apatite maturation. Incorporation of BAG into a self-etch adhesive (Clearfil SE Bond) preserved long-term bond strength, enhanced dentin microhardness, promoted mineral precipitation at the adhesive interface, and improved hybrid layer, sealing—particularly in artificially demineralized dentin.

## Introduction

Dental caries is the most prevalent pathological alteration of dentin [1]. Within this substrate, caries lesions can be classified according to dentin consistency into soft, leathery, firm, and hard dentin [2, 3]. Soft dentin is composed of necrotic, softened, and irreversibly damaged tissue. Bacterial contamination leads to proteolytic degradation of the organic matrix and acidic demineralization of the inorganic phase [4]. In contrast, leathery dentin is partially demineralized and physiologically capable of remineralization, despite its high porosity, collagen fiber exposure, and decreased surface energy [5, 6]. In this zone, the collagen cross-linking remains intact, providing a scaffold for intertubular dentin remineralization. Therefore, leathery dentin, should be preserved during clinical procedures [7, 8].

Numerous studies have sought to restore mineral content to demineralized dentin to recover its mechanical properties [9–11]. Mineral replenishment within the mineral-depleted dentin matrix can facilitate remineralization of the hybrid layer, potentially yielding tissue more resistant to degradation and hydrolysis [12]. Residual minerals within the intrafibrillar collagen region, after partial dentin demineralization may act as nucleation sites for apatite growth, promoting remineralization and mechanical recovery [13]. Moisture within dentin further favors this process, as water enables calcium phosphate precursors to infiltrate collagen fibrils and initiate apatite crystal nucleation and growth [7].

Ion-releasing bioactive materials represent a promising strategy for promoting calcium and phosphate deposition in hydrated or collagen-exposed regions of the hybrid layer [13]. These materials can also inhibit endogenous matrix metalloproteinases (MMPs) via apatite formation, protecting collagen fibrils from premature degradation [14]. Among them, 45S5 bioactive glass (45S5 BAG)—originally developed for bone tissue engineering—stands out for its biocompatibility, chemical similarity to the natural mineral phase of teeth, and strong ability to induce calcium phosphate precipitation and hydroxyapatite crystallization, particularly in simulated body fluids [15].

Given its proven remineralization potential, incorporating 45S5 BAG into restorative materials offers a strategy to recover dentin properties [16]. Such materials can strengthen the resin-dentin bonds, improve resistance to enzymatic degradation and demineralization, and reduce long-term mineral loss [14, 17–19]. The continuous release of calcium and phosphate ions may also limit biofilm penetration through marginal gaps, reducing secondary caries risk [20], and occlude dentinal tubules through carbonated hydroxyapatite deposition, potentially alleviating dentin hypersensitivity [16, 21].

Previous studies have demonstrated the successful incorporation of 45S5 BAG into both etch-and-rinse and self-etch adhesive systems, resulting in enhanced remineralization of demineralized dentin, improved hybrid layer stability, and increased resistance to enzymatic degradation [7, 13]. A study comparing BAG surface pretreatment with BAG-modified adhesives found that incorporation of BAG into the adhesive resulted in superior long-term bonding performance, highlighting the advantages of continuous ion release from BAG-modified adhesives [22]. Moreover, experimental adhesives containing BAG have shown potential to induce apatite formation at the resin–dentin interface and to reduce dentin permeability while maintaining bond strength [23]. Surface application of BAG in suspension has also been reported in a single study to promote localized mineral deposition and apatite nucleation when in contact with dentin, supporting its potential use as a topical remineralizing strategy [24]. Collectively, these findings emphasize the versatility of BAGs as bioactive agents in restorative dentistry and support their use in both surface treatments and adhesive incorporation approaches. Despite these encouraging findings, most available evidence is based on experimental adhesive formulations or focuses exclusively on demineralized substrates, with limited information regarding the performance of BAG-modified commercial adhesive systems when applied to different dentin conditions. Moreover, to the best of our knowledge, no previous study has simultaneously investigated the effects of BAG incorporation into a self-etch adhesive system on both sound dentin and artificially demineralized dentin, considering not only bonding performance but also interfacial sealing, substrate remineralization, and mechanical recovery. Therefore, this study evaluated the ability of a self-etch adhesive system containing 45S5 BAG to induce mineral formation in sound and artificially demineralized dentin and to improve the quality and durability of the adhesive interface. The null hypothesis was that BAG incorporation would not: (1) improve bond strength to sound or demineralized dentin; (2) restore the surface microhardness of demineralized dentin.

## Materials and methods

### Experimental design

**Fig. 1:**
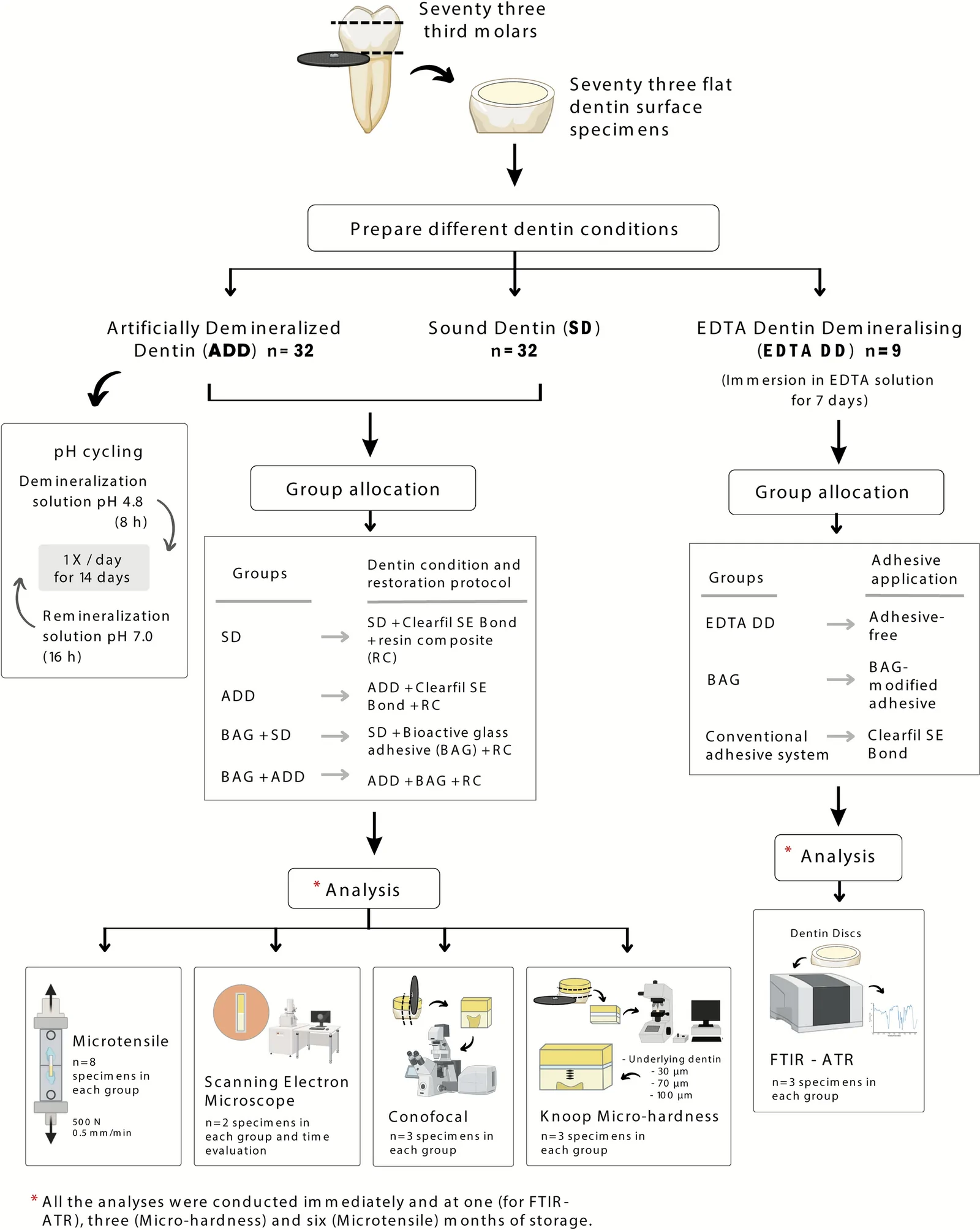
Schematic representation of the experimental design and specimen distribution according to dentin condition, surface treatment, and analytical procedures. Extracted third molars were prepared to obtain flat dentin surfaces and allocated into sound dentin (SD), artificially demineralized dentin (ADD), and EDTA-demineralized dentin (EDTA DD). Specimens were restored using either a conventional self-etch adhesive or a bioactive glass (BAG)–modified adhesive system. Analyses included microtensile bond strength, scanning electron microscopy (SEM), confocal laser scanning microscopy, Knoop microhardness, and FTIR-ATR spectroscopy, performed at different evaluation periods

The independent variable was the incorporation of 10% (w/v) 45S5 bioactive glass (BAG) into a commercial self-etch adhesive system, applied to both sound dentin and artificially demineralized dentin. Evaluations were conducted at two time points: immediately after application and after six months of storage. The dependent variables were bond strength (n = 8) and surface microhardness (n = 3), quantitatively assessed using the microtensile bond strength test and Knoop hardness measurements, respectively. The adhesive interface was also qualitatively analyzed through nanoleakage evaluation (n = 2), and dentin micropermeability assessment (n = 3). Additionally, Fourier-transform infrared spectroscopy (FTIR) (n = 3) was analyzed in demineralized dentin disc treated with the tested adhesives (Fig. 1).

### Bioactive glass preparation

A commercial BAG (45S5, Sigma Aldrich, St. Louis, USA) was used. Prior to incorporating into the adhesive system, the glass was ground and sieved through a series of mesh sizes (150 μm, 75 μm, 53 μm, 38 μm, 20 μm; Cial Paulínia, São Paulo, Brazil). Only particles passing through the 20 μm sieve were selected for use [25].

### Incorporation of bioactive glass into the adhesive system

The self-etch adhesive system Clearfil SE Bond (Kuraray Medical Inc., Tokyo, Japan) was used for BAG incorporation at a concentration of 10% (w/v) [7]. The required mass of BAG was weighed on an analytical balance and added to the adhesive bond component. The mixture was first homogenized using a vortex mixer (QL-901, Biomixer, São Paulo, Brazil) and then subjected to ultrasonic agitation for 1 h to achieve uniform particle dispersion. All procedures were performed at room temperature (25 ℃), under yellow light to avoid premature polymerization [13].

### Preparation of artificially demineralized dentin (ADD)

Sixty-four sound third molars were collected with institutional ethical approval and stored in 0.01% thymol solution at 4 °C. Each tooth was sectioned to obtain a flat mid-coronal dentin surface using a low-speed diamond saw under water cooling (Isomet 4000; Buehler, Lake Bluff, USA), removing the occlusal enamel and roots. The exposed dentin surfaces were polished with 600-grit silicon carbide paper under distilled water for 30 s to create a standardized smear layer.

Half of the specimens underwent a pH cycling protocol to produce artificially demineralized dentin. Teeth were coated with acid-resistant varnish, leaving only the occlusal dentin surface exposed. The demineralization solution contained 1.5 mmol/L CaCl₂, 0.9 mmol/L KH₂PO₄, 50 mmol/L acetic acid, and 5 mmol/L NaN₃, adjusted to pH 4.8. The remineralization solution contained 1.5 mmol/L CaCl₂, 0.9 mmol/L NaH₂PO₄, 0.13 mmol/L KCl, and 5 mmol/L NaN₃, adjusted to pH 7.0 with a HEPES buffer. Each cycle consisted of immersion in 10 mL of the demineralization solution for 8 h, followed by 16 h in the remineralization solution, using fresh solutions daily. The cycle was repeated for 14 days [26].

### Microtensile bond strength test (μTBS) and failure mode analysis

Teeth were randomly allocated into four groups (n = 8) according to adhesive protocol and dentin condition: sound dentin (SD), artificially demineralized dentin (ADD), BAG-modified adhesive on sound dentin (BAG + SD), and BAG-modified adhesive on demineralized dentin (BAG + ADD). The primer was actively applied for 20 s, gently air-dried for 5 s, and left undisturbed for 2 min. The adhesive was then applied, air-thinned for 5 s, and light-cured for 40 s (Bluephase; Ivoclar Vivadent, Schaan, Liechtenstein). A microhybrid composite resin (Filtek Z100; 3 M ESPE, St. Paul, MN, USA) was placed in five 1 mm increments, each light-cured for 40 s at 1200 mW/cm^2^.

Specimens were stored in artificial saliva (1.5 mM CaCl₂, 0.9 mM NaH₂PO₄, 0.13 M KCl, 5 mM NaN₃, HEPES-buffered, pH 7.0) at 37 °C for 24 h, then sectioned into beams (0.9 mm × 0.9 mm cross-section) using a diamond saw. Half of the beams were tested immediately under tension in a universal testing machine (Instron 3345; Canton, MA, USA) with a 500 N load cell at a crosshead speed of 0.5 mm/min. The remaining beams were stored in artificial saliva for six months before testing. The exact cross-sectional area of each beam was measured with a digital caliper, and μTBS values were expressed in MPa. Fracture surfaces were examined under a stereomicroscope at 80 × magnification (SZ 40–50; Olympus, Tokyo, Japan) and classified as: adhesive, cohesive in resin composite, cohesive in dentin or mixed failures (simultaneous occurrence of adhesive and cohesive failures).

### Nanoleakage evaluation

Two teeth per group were restored, and in each evaluation period (immediate and after 6 months of aging), two sticks were selected for nanoleakage evaluation. Each specimen was individually immersed in an aqueous ammoniacal silver nitrate solution (50% wt; pH 7.0) and stored in the dark for 24 h. After rinsing, the beams were placed in a photo-developing solution (Kodak, Rochester, NY, USA) for 8 h under fluorescent light to reduce the silver ions [27]. Subsequently, the specimens were thoroughly rinsed with distilled water, embedded in epoxy resin, and sequentially polished using wet silicon carbide papers (#600, #1200, #2000, and #2500), followed by polishing with 3-, 1-, and 0.25-μm diamond pastes (Buehler, Lake Bluff, IL, USA). Ultrasonic cleaning was performed for 5 min between each polishing step. The polished specimens were dehydrated under vacuum for 24 h, sputter-coated with gold, and examined under a field-emission scanning electron microscope (Quanta 450 FEG, FEI, Hillsboro, OR, USA) in backscattered electron mode at 3000 × and 5000 × magnification.

### Confocal micropermeability evaluation (CLSM)

For micropermeability analysis, three teeth per group were restored using adhesives doped with 0.1 wt% Rhodamine B. Specimens were immersed in 0.3% sodium fluorescein under simulated pulpal pressure (15 cm H₂O) for 3 h. After rinsing and ultrasonic cleaning, the teeth were sectioned into resin–dentin slices and polished with ascending SiC papers (#1200–4000). Interfaces were examined by confocal laser scanning microscopy (LSM 710, Zeiss). Fluorescein was excited at 488–495 nm (emission ~ 520 nm), and Rhodamine B at ~ 540 nm (emission ~ 590 nm). Z-stack images were acquired at 1 µm intervals (up to 10–12 µm depth) with 1024 × 1024-pixel resolution. Five optical sections were randomly captured from each interface for qualitative micropermeability assessment [15].

### Dentin hardness

Knoop microhardness was evaluated using a microhardness tester (FM 100, Future Tech, Tokyo, Japan) equipped with a Knoop diamond indenter, applying a 10 g load for 30 s. Three teeth per group were restored and sectioned into 2-mm-thick mesiodistal slices, which were subsequently polished with SiC papers (800–4000 grit), followed by felt and diamond paste polishing; ultrasonic cleaning was performed between each step. Hardness measurements were obtained along three independent lines per slice, positioned perpendicularly to the hybrid layer and extending toward the pulp. Indentations were performed at standardized depths of 30 μm, 70 μm, and 100 μm from the hybrid layer. Assessments were carried out at two time points: 24 h and 3 months of storage in artificial saliva [28, 29].

### Fourier transform infrared spectroscopy (ATR-FTIR)

Three teeth per group were selected and sectioned into dentin discs (5 mm in diameter × 1.5 mm in thickness) obtained from extracted human third molars and fully demineralized in 0.5 M EDTA (pH 7.4) for 7 days at 25 °C. Three discs per group (demineralized dentin, adhesive, and BAG-modified adhesive) were prepared. Adhesive infiltration was performed under constant agitation at 25 °C in darkness for 2 h (1 h with primer and 1 h with adhesive). Excess adhesive was removed by air-thinning for 5 s per side, followed by light-curing for 40 s at 500 mW/cm^2^. Attenuated total reflectance–Fourier transform infrared spectroscopy (ATR-FTIR; IRPrestige21 FTIR-8400S, Shimadzu, Kyoto, Japan) was used to assess the chemical characteristics of the surface of the demineralized dentin discs.

### Statistical analysis

Data normality was assessed using the Kolmogorov–Smirnov test. As normal distribution was confirmed (p > 0.05), two-way ANOVA was applied for microtensile bond strength, and repeated measures ANOVA was used for Knoop microhardness. Pairwise group comparisons were performed using Tukey’s test (SigmaPlot 14.0, Chicago, IL, USA) at a significance level set at 5% (p < 0.05). Premature failures, defined as specimens that failed before being subjected to tensile loading, were recorded and not included in the analysis.

## Results

### Microtensile bond strength (μTBS) and failure mode analysis

**Fig. 2:**
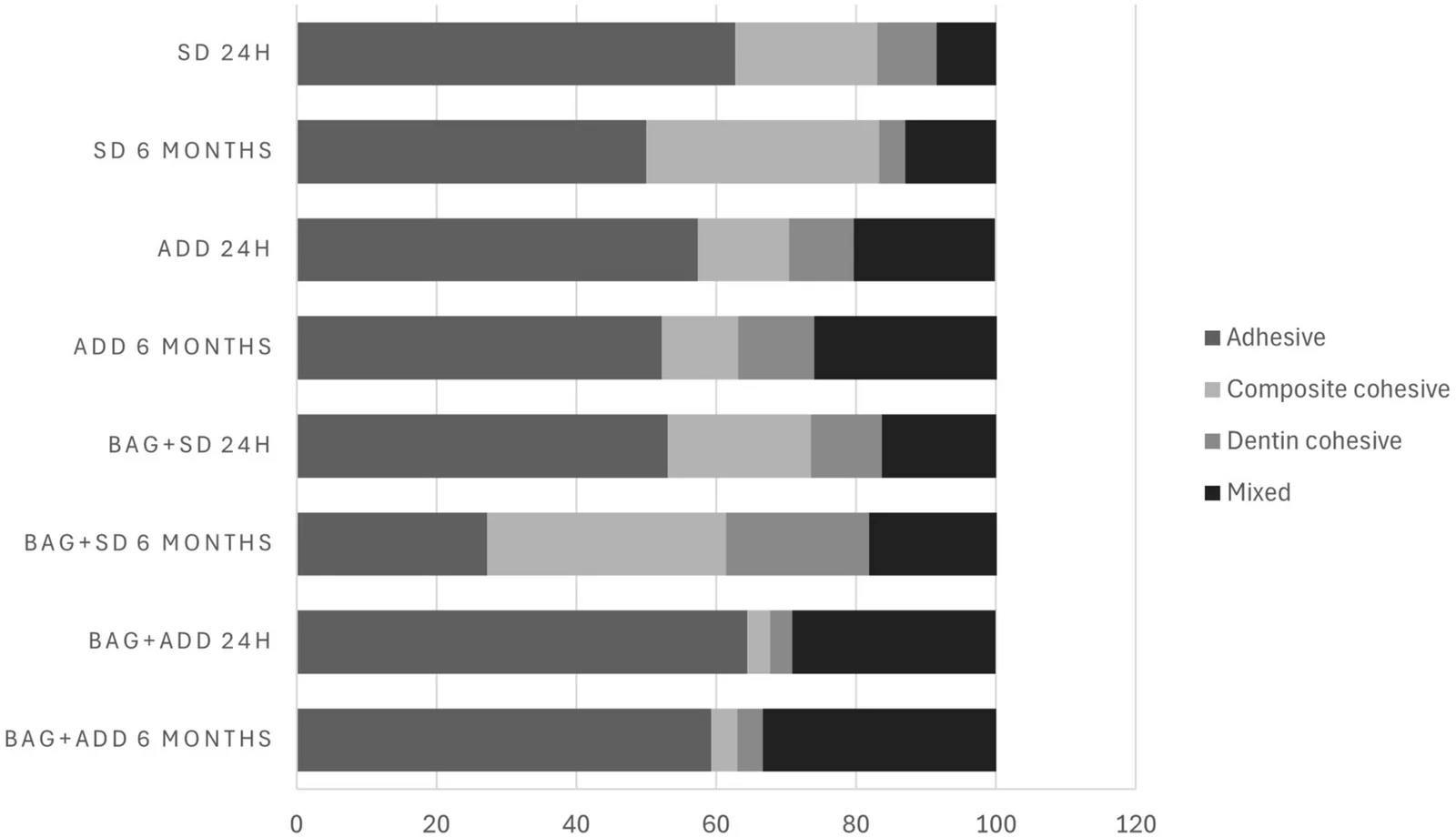
Relative frequency (%) of failure modes in dentin specimens from each group, tested immediately and after 6 months of storage

After 24 h of storage, μTBS values (Table 1) showed no statistically significant differences among the experimental groups. Following 6 months of aging, SD, BAG + SD, and BAG + ADD groups exhibited statistically similar results, all significantly higher than those of the ADD group. When analyzed over time, only BAG + SD and BAG + ADD maintained their bond strength values after 6 months. Across all groups and time points, adhesive failures were the most frequent, regardless of BAG incorporation, with no significant differences in fracture pattern distribution (Fig. 2).

**Table 1:** Mean values (standard deviation) of bond strength, in MPa

	Immediate	6 Months
SD	34.0(8.8) Aa	25.4(5.5) Ab
ADD	32.4(6.2) Aa	15.5(5.8) Bb
BAG + SD	36.4(6.8) Aa	31.5 (4.7) Aa
BAG + ADD	26.41 (3.7) Aa	25.9 (5.3) Aa

Different uppercase letters indicate statistical differences within columns (p < 0.05). Different lowercase letters indicate statistical differences within rows (p < 0.05)

### Nanoleakage

**Fig. 3:**
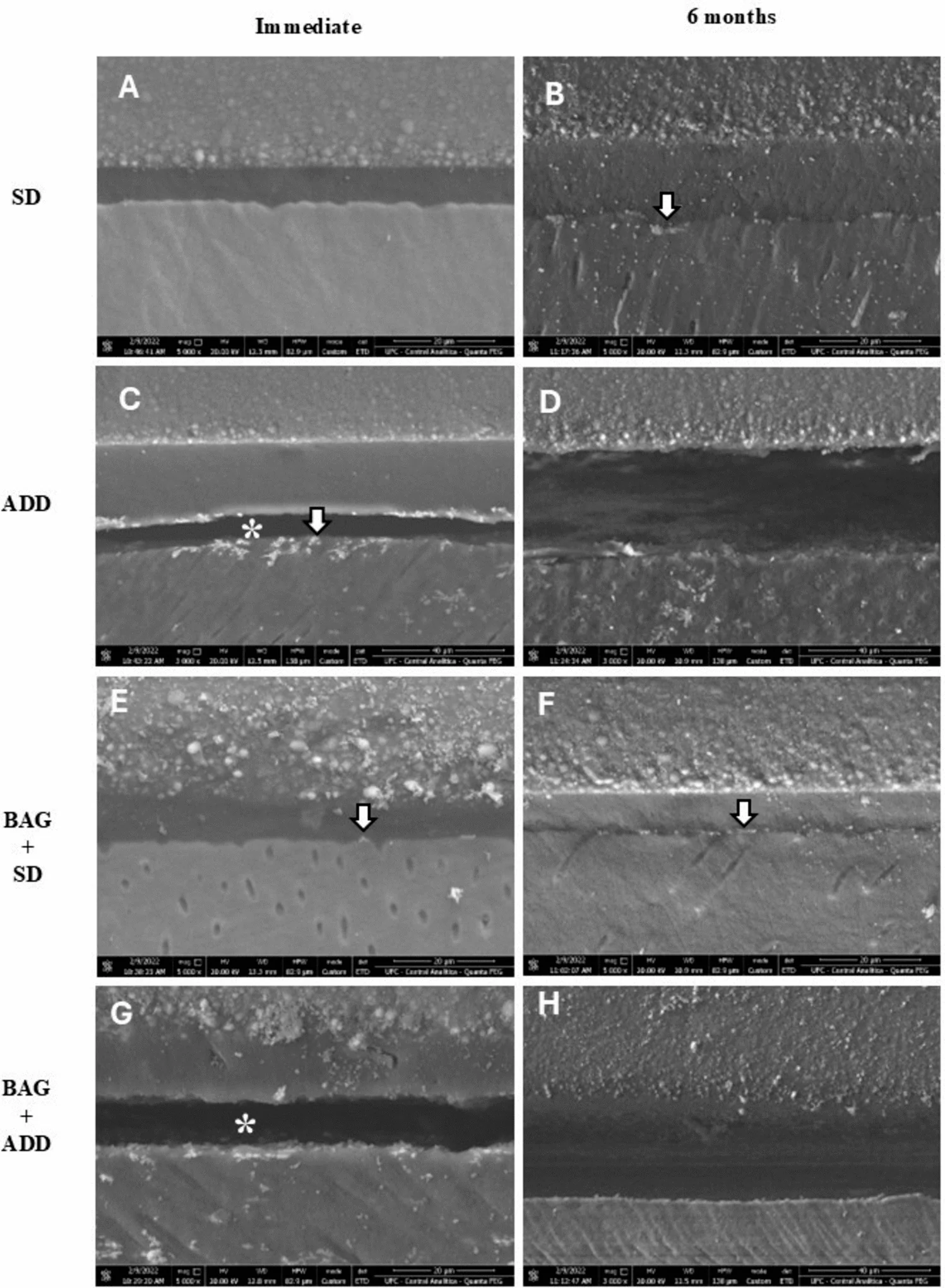
Representative backscattered SEM images of nanoleakage patterns at the resin–dentin interface of sound dentin (SD) and artificially demineralized dentin (ADD), treated with Clearfil SE Bond with or without bioactive glass (BAG), evaluated immediately and after 6 months of aging. **A**, **B** SD: immediately (**A**), the adhesive interface shows minimal silver deposition; after 6 months (**B**), silver uptake along the hybrid layer is indicated by arrows, suggesting increased nanoleakage over time. **C**, **D** ADD: immediately (**C**), intense silver accumulation beneath the hybrid layer (arrow) and interfacial discontinuities (*) are observed; after 6 months (**D**), silver deposition remains evident along the interface. **E**, **F** BAG + SD: immediately (**E**), a well-preserved interface with limited silver deposition is observed; after 6 months (**F**), localized silver uptake can be detected, without evidence of extensive nanoleakage. **G**, **H** BAG + ADD: immediately (**G**), the interface shows a gap; after 6 months (**H**), no evident silver penetration or water-tree formation is observed, indicating greater interfacial stability

In the nanoleakage assessment, sound dentin (SD) specimens (Fig. 3A, B, E and F) showed good preservation of the adhesive interface at baseline, regardless of BAG incorporation. After six months of aging, both SD and BAG + SD groups exhibited silver deposition along the adhesive interface, indicating increased nanoleakage over time. In artificially demineralized dentin (Fig. 3C, D, G and H), the substrate morphology favored crack formation. In artificially demineralized dentin (ADD) (Fig. 3C, D, G and H), the altered substrate morphology favored interfacial defects. The ADD group showed intense silver accumulation beneath the hybrid layer and interfacial discontinuities, which persisted after six months. In contrast, in the BAG + ADD group, a gap associated with intense silver accumulation was observed at baseline; however, after six months, no evident silver penetration or water-tree formation was detected, indicating improved interfacial stability over time.

### Micropermeability

**Fig. 4:**
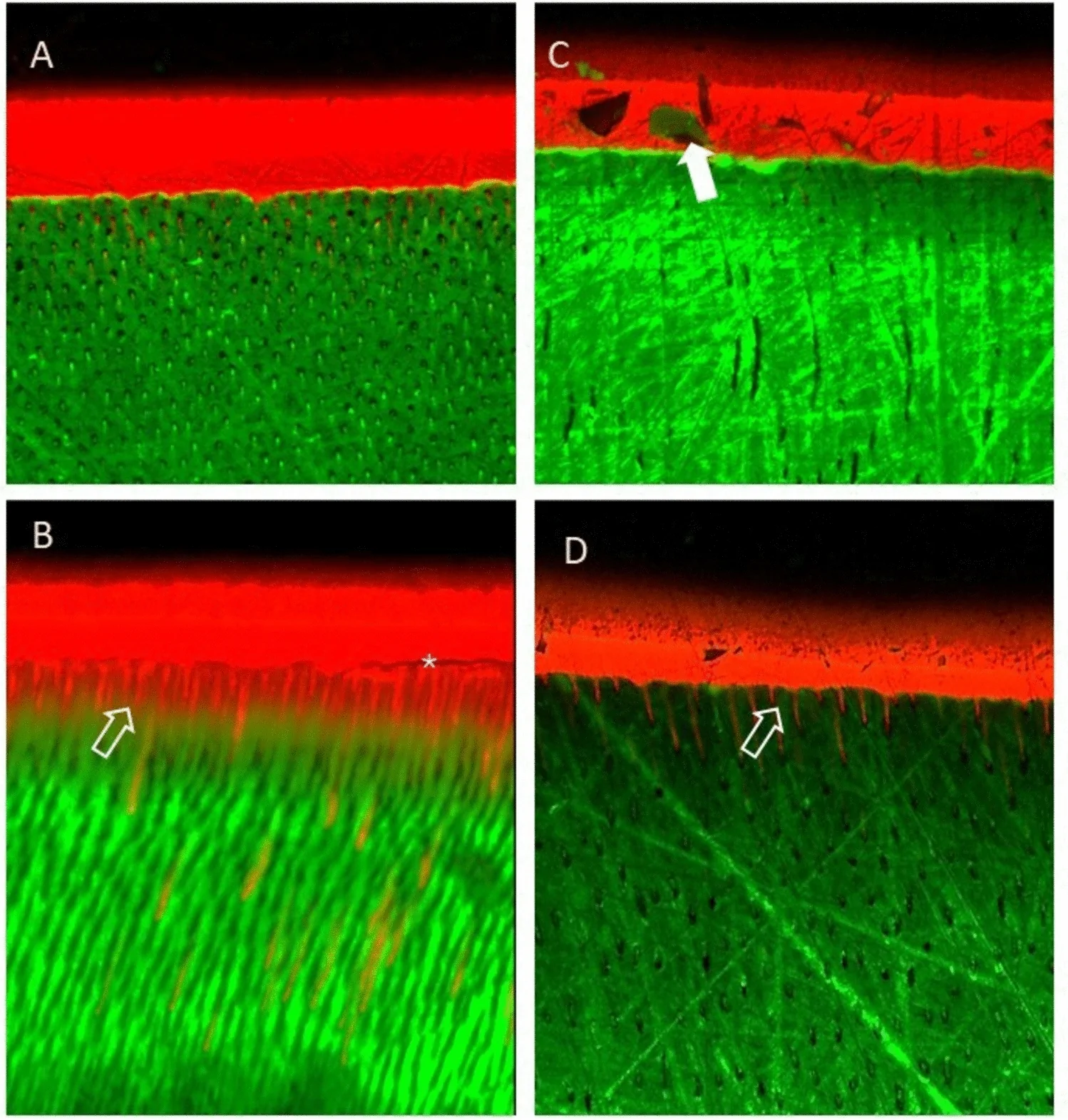
Confocal laser scanning microscopy of dentin-adhesive interfaces. **A** SD and **C** BAG + SD groups showing no detectable micropermeability; closed arrows in **C** indicate BAG particles incorporated into the adhesive layer. **B** ADD group exhibiting intense fluorescein penetration throughout the hybrid layer and adjacent dentin, with a distinct interfacial gap (*); open arrows indicate fluorescein infiltration. **D** BAG + ADD group showing reduced fluorescein infiltration and a more homogeneous interface, suggesting improved sealing ability

Confocal microscopy revealed marked fluorescein micropermeability, characterized by intense dye penetration throughout the hybrid layer and adjacent dentin, in the ADD group (Fig. 4B), where a distinct interfacial gap (*) was observed. Open arrows indicate fluorescein infiltration. In contrast, the BAG + ADD group (Fig. 4D) demonstrated substantially reduced fluorescein infiltration, with a more homogeneous interface and fewer structural disruptions, suggesting improved sealing ability. In sound dentin, neither the DH (Fig. 4A) nor the BAG + DH (Fig. 4C) groups exhibited detectable micropermeability; however, closed arrows indicate the presence of BAG particles within the adhesive layer in Fig. 3C, confirming their incorporation without compromising interfacial integrity.

### Knoop hardness (KH)

KH was evaluated at different depths. At baseline, SD and BAG + SD showed significantly higher values than other groups, regardless of depth. After three months of storage in artificial saliva, BAG + ADD presented increased KH at all depths, while BAG + SD improved hardness at 70 µm and 100 µm (p = 0.006 and p = 0.015, respectively; Table 2).

**Table 2:** Mean Knoop microhardness (MK) values at different dentin measurement depths (underlying, 30μm, 70μm, 100μm) and evaluation times (immediate and after 3 months)

Measurement depths	Evaluation times	Experimental groups			
		SD	ADD	BAG + SD	BAG + ADD
Underlying dentin	Immediate	47.5 (7.6) Aa	1.2 (1.5) Ab	54.1 (8.2) Aa	8.7 (8.2) Bb
	3 months	47.2 (11.2) Ab	2.2 (3.0) Ad	58.3 (10.3) Aa	17.6 (9.9) Ac
30μm	Immediate	54.2 (7.2) Aa	3.4 (2.0) Bc	53.8 (7.4) Aa	17.4 (10.0) Bb
	3 months	52.8 (15.9) Aa	7.5 (7.1) Ac	58.9 (10.8) Aa	29.0 (8.9) Ab
70μm	Immediate	53.9 (9.2) Aa	8.7 (4.8) Ac	53.1 (9.2) Ba	26.6 (7.5) Bb
	3 months	52.4 (12.4) Ab	12.7 (6.6) bAd	63.5 (9.2) Aa	37.1 (9.7) Ac
100μm	Immediate	56.5 (7.3) Aa	19.3 (6.8) Ac	53.6 (8.9) Ba	35.1 (7.4) Bb
	3 months	53.4 (14.3) Ab	20.4 (5.5) Ac	63.4 (12.5) Aa	44.9 (8.5) Ab

Different uppercase letters in each dentin depth indicate statistical differences within columns (p < 0.05). Different lowercase letters in each dentin depth indicate statistical differences within rows (p < 0.05)

### Fourier transform infrared spectroscopy (FTIR-ATR)

**Fig. 5:**
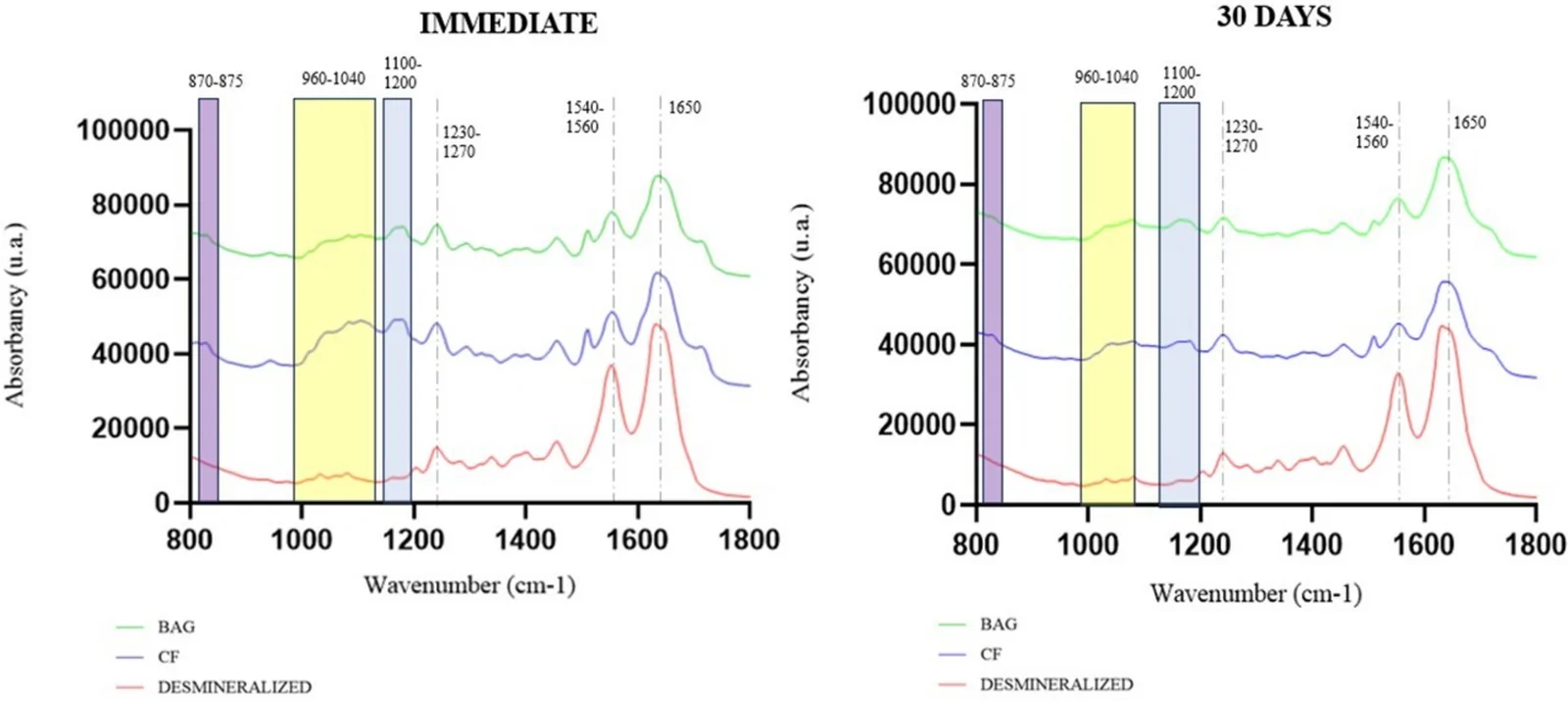
FTIR-ATR spectra of dentin treated with Clearfil SE Bond (CF) or Clearfil SE Bond containing bioactive glass (BAG), evaluated immediately and after 30 days. The highlighted regions indicate carbonate (~ 870–875 cm⁻^1^), phosphate (~ 960 and ~ 1040 cm⁻^1^; 1100–1200 cm⁻^1^), and organic matrix bands, including Amide III (1230–1270 cm⁻^1^), Amide II (1540–1560 cm⁻^1^), and Amide I (~ 1650 cm⁻^1^). Spectral overlap between adhesive resin, bioactive glass, and dentinal components may partially mask phosphate-related vibrations, whereas phosphate bands are more clearly observed in demineralized dentin after 30 days

FTIR-ATR analysis (Fig. 5) revealed distinct spectral profiles among the experimental groups and evaluation periods. In the BAG-containing group, phosphate-related bands (~ 960, ~ 1040 and 1100–1200 cm⁻^1^) and the carbonate band (~ 870–875 cm⁻^1^) were detected at both time points; however, these signals partially overlapped with contributions from both the adhesive resin and bioactive glass components, which may have masked phosphate vibrations associated with dentinal hydroxyapatite. After 30 days, increased band definition was observed, suggesting time-dependent ion release and maturation of carbonated apatite. In the Clearfil SE Bond group, mineral-related bands were less intense and largely masked by the resin matrix at both evaluation periods. In contrast, demineralized dentin exhibited reduced phosphate-related bands and pronounced organic matrix signals, including Amide III (1230–1270 cm⁻^1^), Amide II (1540–1560 cm⁻^1^), and Amide I (~ 1650 cm⁻^1^). Notably, after 30 days, phosphate-related bands (~ 960 and ~ 1040 cm⁻^1^) became more evident in demineralized dentin due to the absence of spectral overlap from bioactive glass or resin components.

## Discussion

Bioactive glasses (BAGs) have been incorporated into dental materials to confer bioactivity through ion release, promoting hydroxyapatite formation [30]. Several studies have shown that this addition can provide benefits to a variety of dental materials, such as gutta-percha, adhesive systems, endodontic cements, protective materials, toothpastes, composite resins, and self-adhesive resins [16, 31]. Despite promising results, their ability to maintain a stable adhesive interface remains under investigation [32]. In the present study, Clearfil SE Bond was selected as the reference adhesive due to its well-documented clinical performance, predictable behavior, and stable interaction with hydroxyapatite mediated by the functional monomer 10-methacryloyloxydecyl dihydrogen phosphate (10-MDP), allowing assessment of BAG incorporation in a clinically relevant system [33, 34].

The present study demonstrated that incorporating 10% BAG into a commercial self-etch adhesive system yielded microtensile bond strength values comparable to Clearfil SE Bond after 24 h, even when applied to artificially demineralized dentin. After six months of storage, however, significantly lower values were recorded for the SD and ADD groups, whereas all BAG-containing groups maintained their initial bond strength. These findings fail to reject the first hypothesis. Similarly to Carvalho et al., who reported only minor bond strength reductions after one year in BAG-containing experimental self-etch adhesives, in contrast to marked declines in Clearfil SE Bond and BAG-free controls [32].

The reduced long-term performance of the SD group may be related to the mild acidity of the adhesive, which likely resulted in limited resin penetration into the substrate. This shallow infiltration may accelerate degradation of the adhesive interface [35]. The bonding effectiveness of self-etch adhesives is known to be influenced by the smear layer, particularly when using mild or ultra-mild formulations [36]. Because the hybrid layer morphology depends on the pH of the primer, mild adhesives produce only superficial demineralization, resulting in submicron hybrid layers [37]. In addition, the low pH of self-etch adhesives can activate matrix metalloproteinases (MMPs), leading to collagen degradation within inadequately infiltrated zones [38], ultimately compromising hybrid layer integrity and bond durability [39]. In contrast, BAG-containing groups maintained stable bond strength over time, likely due to the alkaline nature of BAG, which can neutralize the adhesive’s acidity, reduce MMP activation, and minimize collagen breakdown [40]. Within this context, it is important to consider that the adhesive system used in this study is a mild two-step self-etch adhesive containing 10-MDP, a functional monomer capable of establishing stable chemical bonds with calcium in residual hydroxyapatite. In addition to micromechanical interlocking, 10-MDP-based adhesives promote the formation of a chemically stable acid–base–resistant zone and self-assembled nanolayers at the resin–dentin interface, which have been associated with enhanced bond durability and resistance to hydrolytic degradation. However, in the absence of BAG, these chemical bonding mechanisms alone may not be sufficient to counterbalance the effects of limited infiltration and enzymatic activity over time, particularly in challenging substrates. These intrinsic properties of the adhesive may have contributed to the favorable bonding performance observed in the current study. However, the  improved long-term bond strength preservation observed in the BAG-modified groups therefore suggests an additive or synergistic effect between bioactive glass incorporation and the chemical bonding potential of 10-MDP. The sustained release of calcium and phosphate ions from bioactive glass may further stabilize the adhesive interface by promoting mineral deposition within water-filled regions of the hybrid layer and reinforcing the resin–dentin bond over time.

In addition to the bioactive effects promoted by BAG incorporation, adhesive performance may also be influenced by the concentration of BAG particles within the resin matrix. In the present study, a 10% BAG concentration was selected based on previous reports [7]; however, it is important to acknowledge that BAG loading can significantly affect the rheological properties and viscosity of adhesive systems [41]. Higher filler contents may increase viscosity and potentially hinder resin infiltration into the dentin substrate, whereas lower concentrations may reduce ion-release capacity and compromise bioactivity [42]. Therefore, future studies should investigate the influence of BAG concentration on adhesive rheology, monomer diffusion, hybrid layer formation, and long-term bonding stability.

Another advantage observed was the lower nanoleakage and greater sealing integrity in the BAG + ADD group after aging, unlike the SD group, which exhibited intense silver deposition below the hybrid layer and increased leakage over time. The stability of the BAG + ADD group suggests that mineral precipitation may have occurred within voids, displacing water and reinforcing the interface. Bertassoni et al. demonstrated that continuous calcium (Ca^2^⁺) and phosphate (PO₄^3^⁻) ion release can interact with the dentin organic matrix, promoting remineralization and mechanical recovery [41]. Similarly, the ion-exchange between BAG and the storage medium favors mineral deposition at the adhesive interface, potentially explaining the reduced nanoleakage and preserved bond strength [40]. Biomimetic remineralization processes can gradually replace residual water-filled spaces within the hybrid layer with apatite crystals formed from amorphous calcium phosphate nanoprecursors [42].

In minimally invasive dentistry, restoring caries-affected dentin has become an accepted option [43]. However, adhesion to this substrate remains challenging due to its thick, porous hybrid layer [5], constricted or obliterated dentinal tubules [8], and the imbalance between reduced mineral content and increased degradation of collagen and apatite crystals by bacterial enzymes [29]. Since this zone often represents the main bonding interface [42], there is a strong rationale for developing materials that can improve adhesion to caries-affected dentin. Recent studies highlight multiple benefits of BAG incorporation into dental materials [29]. Beyond releasing calcium, phosphate, and fluoride ions to promote remineralization, BAG can inhibit bacterial growth without affecting pulp vitality or the mechanical performance of composites [7, 39]. BAG particles have also been shown to preserve collagen fibril integrity within the hybrid layer after aging [13]. These effects align with our results, in which BAG preserved substrate characteristics and recovered mechanical properties in both sound and demineralized dentin.

Ultramorphological evaluation via micropermeability testing further revealed that BAG improved interface sealing. In demineralized dentin, BAG application reduced fluorescein penetration in the hybrid layer compared to untreated controls. Sauro et al. similarly reported reduced micropermeability along the resin–dentin interface in adhesives containing BAG [15]. This protective effect may be linked to hydrated silica (Si(OH)₄) release upon BAG contact with body fluids, which can diffuse and bind nonspecifically to exposed collagen [44]. The ionic dissolution products from BAG may activate alkaline phosphatase, reduce collagen degradation, enhance extracellular matrix production, and promote mineralization [39], thereby protecting the hybrid layer and reducing endogenous MMP activity [43].

Hardness is often reduced in caries-affected dentin compared with sound dentin, making its recovery clinically important. In the present study, BAG significantly increased the microhardness of artificially demineralized dentin at all depths over time. However, BAG + ADD values at 100 μm depth were statistically similar to those of the SD group. This way, the outcomes reject the second hypothesis. BAG’s alkalinizing effect on the incubation medium may reduce solubilization of dentin collagen telopeptides and improve the stiffness of demineralized matrices [19]. These findings agree with Carneiro et al., who observed similar results when incorporating niobiophosphate BAG into a commercial adhesive [13], and with Abuna et al., who reported increased microhardness in caries-affected dentin treated with a nanobioactive glass-containing self-etch adhesive [7]. BAG may release Si(OH)₄, which binds to demineralized collagen and polymerizes into a SiO₂-rich layer [45]. This layer promotes amorphous calcium phosphate precipitation and its transformation into non-stoichiometric apatite [46], while the resulting alkaline environment supports Si(OH)₄ condensation and Ca^2^⁺/PO₄^3^⁻ precipitation, inactivating MMPs and cathepsins and reducing proteolytic activity [47]. It should be noted, however, that microhardness measurements provide only indirect evidence of remineralization, reflecting changes in surface stiffness rather than quantifying mineral deposition or structural recovery throughout the dentin matrix. Therefore, microhardness results must be interpreted with caution and in conjunction with complementary morphological and chemical analyses [48].

FTIR-ATR analysis revealed distinct spectral profiles among the experimental groups and evaluation periods, reflecting differences in mineral and organic contributions at the dentin surface. In the BAG-containing groups, phosphate-related bands at approximately 960 and 1040 cm⁻^1^, a broad contribution in the 1100–1200 cm⁻^1^ region, and a carbonate band at ~ 870–875 cm⁻^1^ were detected at both time points. These features are consistent with the formation of calcium phosphate phases associated with carbonated apatite precursors. However, their interpretation must consider spectral overlap with vibrations from the resin matrix and, in particular, with the broad Si–O–Si stretching bands of the silica-rich 45S5 bioactive glass, which can partially mask phosphate ν₁ and ν₃ vibrations when calcium phosphate phases are poorly crystalline or amorphous [49, 50].

After 30 days of aging, increased definition of phosphate-related bands was observed in BAG-treated specimens, indicating a time-dependent process associated with sustained ion release and progressive maturation of calcium phosphate phases. This behavior is consistent with the classical bioactivity mechanism of 45S5 bioactive glass, involving initial silica dissolution, formation of a silica-rich layer, and subsequent nucleation of amorphous calcium phosphate followed by gradual transformation into hydroxycarbonate apatite [51, 52]. Similar FTIR patterns, characterized by initially broad phosphate bands that become more defined over time, have been reported during dentin remineralization induced by bioactive glasses, supporting the interpretation that early spectral changes reflect mineral precursors rather than fully crystalline apatite [49, 51]. In the Clearfil SE Bond control group, mineral-related bands were weak and largely masked by the resin matrix at both evaluation periods. In contrast, demineralized dentin exhibited a predominantly organic spectral profile, with pronounced amide bands (Amide III, II, and I) and reduced phosphate signals at the immediate evaluation. After 30 days, phosphate-related bands became more evident in this group, most likely due to reduced spectral overlap rather than mineral deposition [49, 51].

It should be emphasized that the high silica content of 45S5 bioactive glass represents an intrinsic limitation of FTIR for detecting apatite formation, as silicate bands may obscure phosphate vibrations, particularly during early bioactivity stages [50, 52]. Therefore, the absence of sharp apatite bands does not preclude ongoing mineral precursor formation, but reflects the amorphous nature of early reaction products. Overall, these findings support the bioactive potential of BAG-containing adhesive systems, while highlighting the need for complementary mechanical and morphological analyses to confirm remineralization and mineral formation at the adhesive interface.

Remineralization of partially demineralized dentin may restore hardness and mechanical properties; however, adhesive durability on this substrate remains inferior to that of sound dentin [44, 53]. The present findings demonstrate that incorporation of BAG into a self-etch adhesive system improves interfacial stability, microhardness, and sealing performance in both sound and demineralized dentin. Nonetheless, this study is limited by the use of artificially demineralized dentin and in vitro conditions, which do not fully replicate the structural, biochemical, and mechanical complexity of the oral environment. Moreover, the aging period was limited to six months, and alternative application protocols or BAG concentrations were not investigated, highlighting the need for further studies.

Despite these limitations, BAG-containing adhesives maintained stable microtensile bond strength over time, even in demineralized dentin, outperforming the conventional system. The ionic dissolution products released from BAG may contribute to long-term interfacial stability by inhibiting proteolytic activity and preserving collagen integrity, although these mechanisms require further clarification [7]. Ultramorphological analyses provided initial evidence of improved interfacial sealing, supporting the bioactive potential of BAG-modified adhesives.

It can be concluded that the incorporation of bioactive glass into the the resin component of a self-etch adhesive system effectively preserved dentin bond strength regardless of the substrate and reduced nanoleakage after six months of storage. Moreover, it restored the microhardness of artificially demineralized dentin and promoted the precipitation of mineral precursors.

## Data Availability

Data will be made available on request.
